# Human sulfatase 1 exerts anti-tumor activity by inhibiting the AKT/ CDK4 signaling pathway in melanoma

**DOI:** 10.18632/oncotarget.12996

**Published:** 2016-10-31

**Authors:** Xiaoli Lou, Bin Sun, Jianxing Song, Yicun Wang, Junhao Jiang, Yang Xu, Zeqiang Ren, Changqing Su

**Affiliations:** ^1^ Department of Reconstructive Surgery, Changhai Hospital, Second Military Medical University, Shanghai 200433, China; ^2^ Department of Molecular Oncology, Eastern Hepatobiliary Surgery Hospital and National Center of Liver Cancer, Second Military Medical University, Shanghai 200433, China; ^3^ Department of Orthopedics, Jinling Hospital, Nanjing University Medical College, Nanjing 210002, China; ^4^ Cancer Center for Collaborative Innovation, Affiliated Peixian People's Hospital of Xuzhou Medical University, Jiangsu Peixian 221600, China

**Keywords:** human sulfatase 1 (hSulf-1), protein kinase B (AKT), cyclin-dependent kinase (CDK4), signaling pathway, melanoma

## Abstract

Human sulfatase 1 (hSulf-1) has aryl sulfatase activity. It can reduce the sulfation of cell surface heparan sulfate proteoglycan (HSPG) and inhibit various growth factor receptor-mediated signaling pathways. In most cancers, hSulf-1 is inactivated, which endows cancer cells with increasesed cell proliferation and metastatic activities, inhibition of apoptosis, and decreased sensitivity to radio- and chemotherapy. In this study, we found that hSulf-1 overexpression in melanoma cells can inhibit cell proliferation and induce cell cycle arrest and apoptosis by decreasing the protein kinase B (AKT) phosphorylation and limiting CDK4 nuclear import. We further confirmed that hSulf-1 overexpression can inhibit AKT phosphorylation and CDK4 nuclear localization and retard the growth of melanoma xenograft tumors in nude mice. Overall, hSulf-1 function in melanoma cells provides an ideal molecular treatment target. An important anti-tumor mechanism of hSulf-1 operates by decreasing downstream AKT signaling pathway activity and inhibiting the nuclear import of CDK4.

## INTRODUCTION

The human sulfatase 1 (hSulf-1) gene encodes an aryl sulfatase. hSulf-1 reduces the sulfation of heparan sulfate proteoglycans (HSPGs) in the extracellular matrix and inhibits binding between various cell growth factors and their receptors. It is a negative regulator of cell proliferation. In normal human tissues, hSulf-1 is stably expressed, and it plays important roles in cell proliferation and differentiation [1, 2]. In most cancer cell lines and tumor tissues, hSulf-1 expression levels are markedly decreased. The decreased expression of hSulf-1 is closely related to cancer cell proliferation, increased metastatic activity, inhibition of apoptosis, and reduced sensitivity to radio- and chemotherapy [3–5].

Carcinogenesis is a pathological process that involves multiple genes and multiple stages of development. One important mechanism of carcinogenesis is uncontrolled cell proliferation caused by the excessive activation of cell proliferation signals. On the cell surface and in the extracellular matrix (ECM), HSPGs represent an important class of proteins [6]. They are glycoproteins and are key components of cell-cell and cell-ECM interactions. When the sugar chain of HSPG is in a sulfated state, it has great potential to bind proteins, carbohydrates and other functional biological macromolecules. HSPG can mediate the binding of various cell growth factors to their specific receptors to form receptor signal transduction complexes. HSPG can also activate the activities of receptor tyrosine kinases and thereby regulate cell proliferation, differentiation, adhesion, proliferation and motility [7]. In normal tissues, sulfation and de-sulfation of HSPG are maintained in a relatively balanced state. However, in tumor cells, the sulfation levels of HSPG sugar chains are markedly increased, resulting in the excessive activation of the signal transduction pathways of various growth factors. Increased HSPG sulfation is an important step in malignant progression. In most cancer cells, hSulf-1 expression levels are decreased, which is one reason for increased HSPG sulfation [2, 8]. Therefore, in tumor cells, the hSulf-1 expression level and the HSPG sulfation level are closely related to the activities of various growth factor signaling pathways and the regulation of biological behaviors. They may thus represent important targets for cancer therapy [9].

Our previous studies found that transfection of hSulf-1 in liver cancer cells can inhibit the phosphorylation of protein kinase B (AKT) and extracellular signal-regulated kinase (ERK), thereby blocking the signal transduction pathways of these kinases and inhibiting tumor growth [10, 11]. We further found that hSulf-1 expression in liver cancer cells can reverse bFGF-induced cell cycle progression and promote apoptosis [2]. We also found that hSulf-1 can induce cell cycle arrest in melanoma cells, but the underlying mechanism remained unclear. The present study focuses on hSulf-1 expression in melanoma cells and its relationship with the biological behavior of cancer cells. The molecular mechanisms by which hSulf-1 regulates cell cycle in melanoma cells and melanoma xenografts were also studied.

## RESULTS

### Ectopically expressed hSulf-1 in melanoma cells inhibits cell proliferation

**Figure 1:**
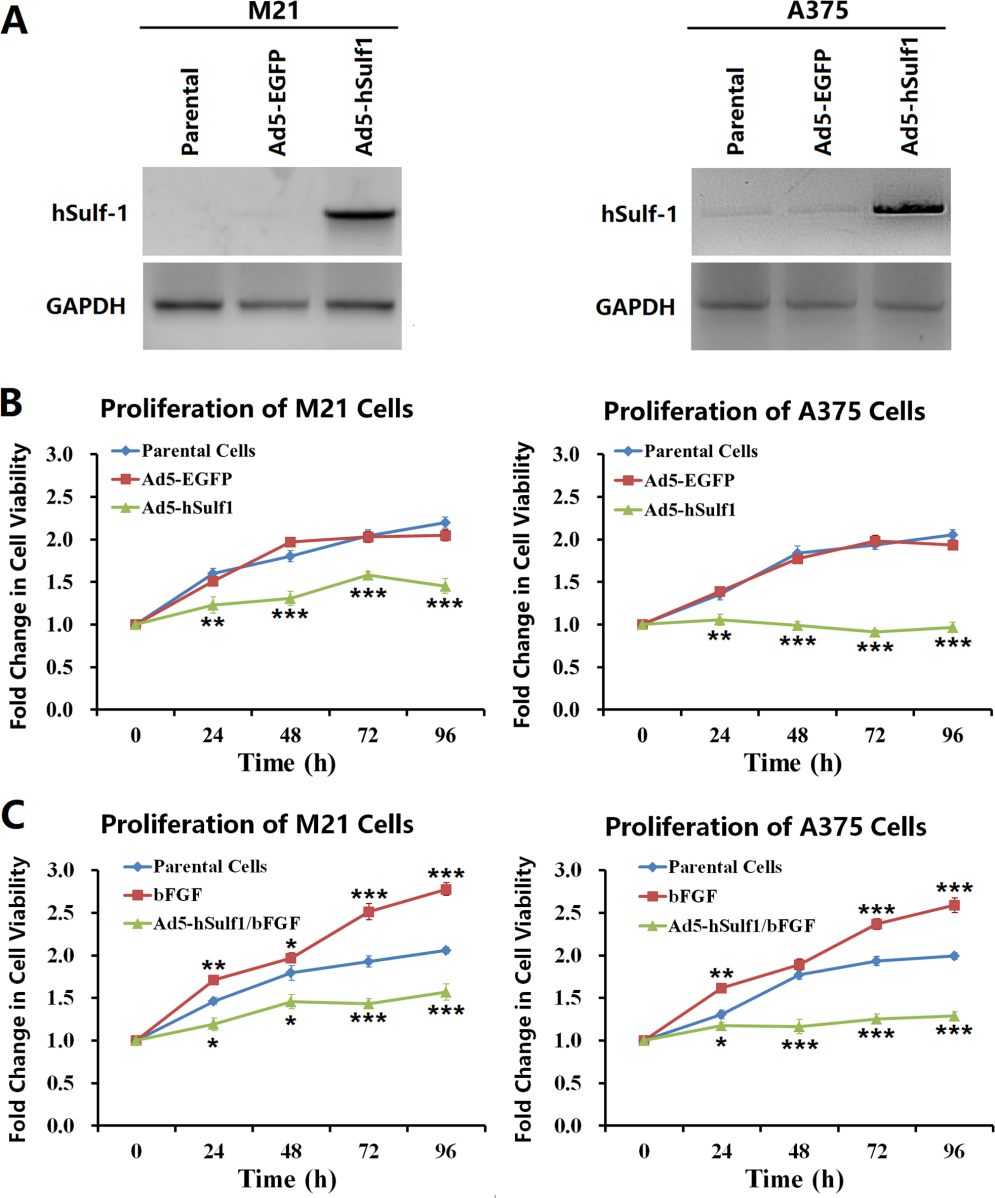
Ectopically expressed hSulf-1 inhibits melanoma cell proliferation (**A**) The melanoma cell lines were planted in 24-well plates at a density of 1 × 10^6^ cells/well for 24 h, then infected with Ad5-hSulf1 or Ad5-EGFP at an MOI of 100 pfu/cell. After continuously cultured for 48 h, cells were harvested and the expression of hSulf-1 was detected by Western blotting. GAPDH was used as the loading control. (**B**) The melanoma cell lines were planted in 96-well plates at a density of 1 × 10^4^ cells/well. After cultured for 24 h, the viruses Ad5-hSulf1 and Ad5-EGFP were used to infect cells at an MOI of 100 pfu/cell. Cell viability was measured by CCK-8 assay at 24 h, 48 h, 72 h and 96 h culture time; **P < 0.01 and ***P < 0.001 compared with the Ad5-EGFP control group at the corresponding timepoint. (**C**) The melanoma cell lines and their virus-infected derivatives in 96-well plates at 1 × 10^4^ cells/well were added bFGF at a final concentration of 20 ng/ml. After 24 h, 48 h, 72 h, and 96 h, cell viability was measured by CCK-8 assay; *P < 0.05, **P < 0.01 and ***P < 0.001 compared with the parental control group at the corresponding timepoint.

The melanoma cell line M21 is negative for hSulf-1 expression, while A375 cells express weakly hSulf-1. When these 2 types of cells were infected with Ad5-hSulf1, hSulf-1 showed strong expression (Figure 1A). By using CCK-8 to evaluate cell proliferation, we found that the cell viability decreased in the derivative cell line M21-hSulf1 overexpressing hSulf-1, while A375-hSulf1 cells showed slower cell proliferation, compared with the negative control group (Figure 1B). The cell proliferation of the M21 and A375 parental cells was enhanced by treatment with basic fibroblast growth factor (bFGF); however, the effects of bFGF on the proliferation of M21-hSulf1 and A375-hSulf1 cells were not obvious (Figure 1C). These results indicate that cell proliferation is inhibited in melanoma cells overexpressing hSulf-1 and that their responses to growth factor are attenuated.

### hSulf-1 expression in melanoma cells induces cell cycle arrest and apoptosis

**Figure 2:**
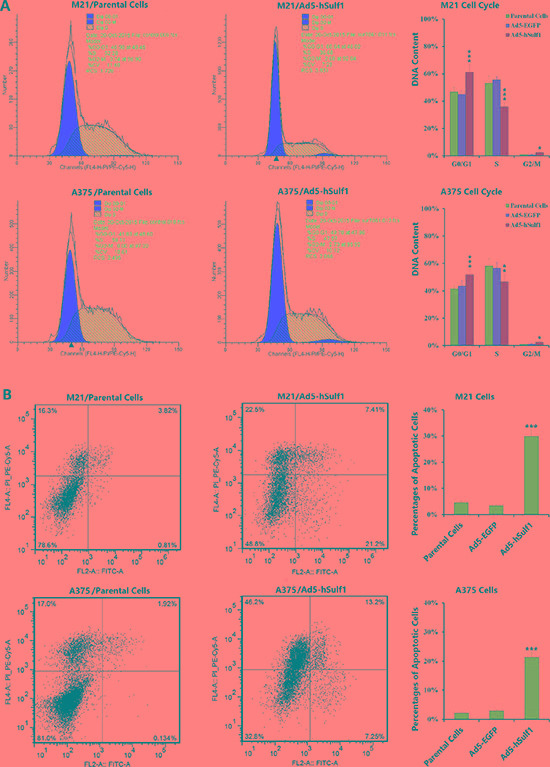
Ectopically expressed hSulf-1 induces melanoma cell cycle arrest and apoptosis (**A**) The melanoma cell lines were planted in 6-well plates at a density of 5 × 10^6^ cells/well for 24 h, then infected with Ad5-hSulf1 or Ad5-EGFP at an MOI of 100 pfu/cell. After continuously cultured for 48 h, cells were harvested and fixed with ice-cold 75% ethanol and incubated in a 4°C overnight. Cells were washed twice in PBS and then incubated with propidium iodide (PI) solution containing RNase for 30 min in dark. Then cell cycle was detected by flow cytometry; *P < 0.05, **P < 0.01 and ***P < 0.001 compared with the Ad5-EGFP control group. (**B**) The above melanoma cell lines and their virus-infected derivatives were stained with Annexin V/PI. Apoptosis was detected with a flow cytometer; ***P < 0.001 compared with the Ad5-EGFP control group.

We used flow cytometry (FCM) to examine cell cycle status and apoptosis. Compared with the negative control group, M21-hSulf1 and A375-hSulf1 cells had increased proportions of cells in G0/G1 phase and decreased proportions of cells in S phase, cells in G2/M phase also had a significant increase (Figure 2A). There are significant increases in apoptosis in the hSulf1-expressing cells (Figure 2B). These results indicate that overexpression of hSulf-1 can induce melanoma cell cycle arrest and apoptosis.

### Cell cycle regulation by hSulf-1 in melanoma cells is associated with the AKT/CDK4 signaling pathway

**Figure 3:**
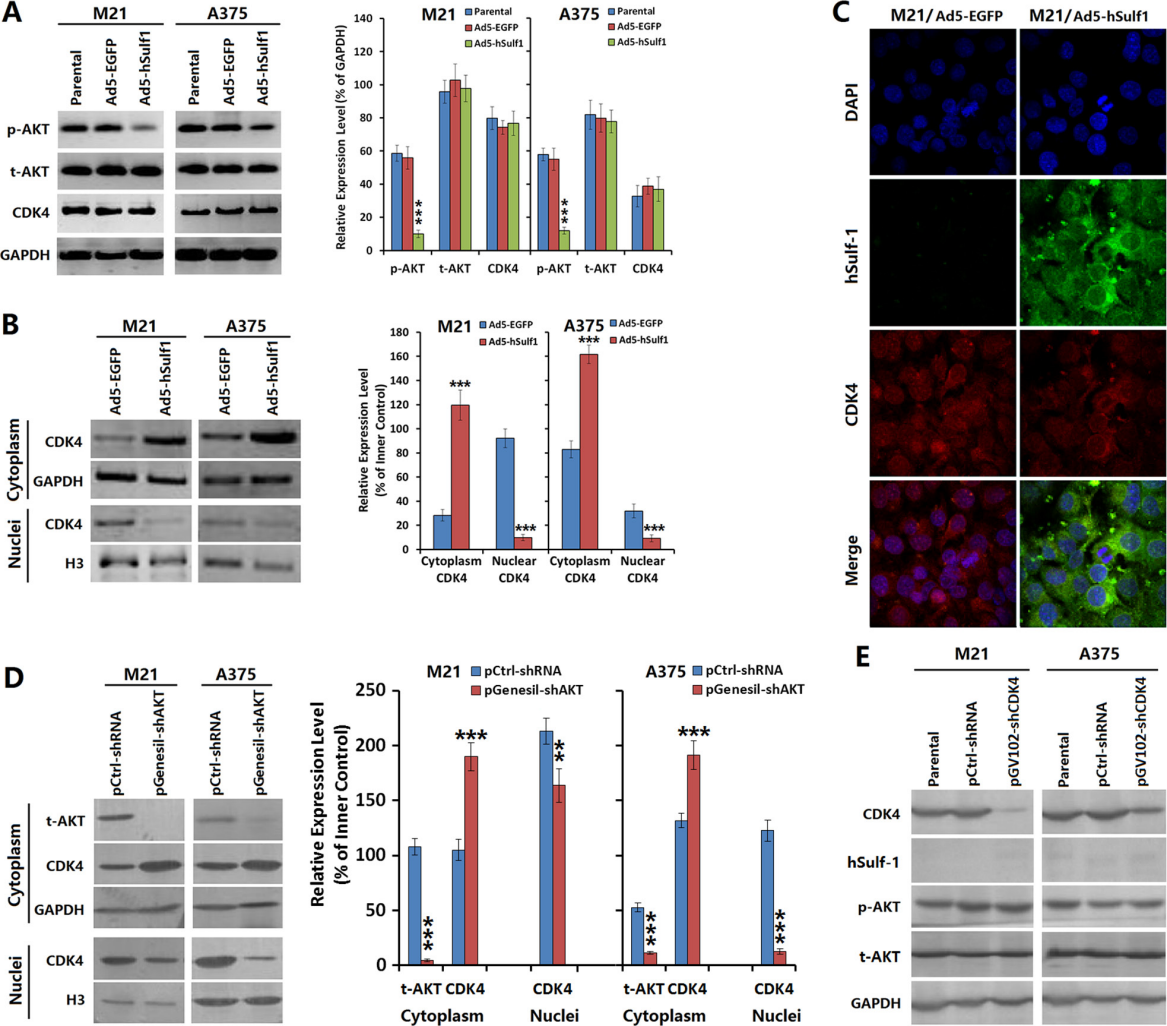
Expression of hSulf-1 in melanoma cells is associated with the AKT/CDK4 signaling pathway (**A**) The melanoma cell lines were planted in 24-well plates at a density of 1 × 10^6^ cells/well for 24 h, then infected with Ad5-hSulf1 or Ad5-EGFP at an MOI of 100 pfu/cell. After continuously cultured for 48 h, cells were harvested and the expressions of p-AKT, t-AKT and CDK4 were detected by Western blotting. GAPDH was used as the loading control. The densitometry analysis of every band was performed normalized with GAPDH content; ***P < 0.001 compared with the Ad5-EGFP control group. (**B**) The above melanoma cell lines and their virus-infected derivatives were collected to extract the cytoplasmic and nuclear proteins and the expression of CDK4 was detected by Western blotting, with GAPDH and histone H3 as the loading control, respectively; ***P < 0.001 compared with the Ad5-EGFP control group. (**C**) The melanoma cells and their virus-infected derivatives were cultured in Lab-Tek chambers at 1 × 10^4^ cells/well. After 24 h, cells were fixed for 30 min in 4% formaldehyde and washed with 0.1 M PBS (pH 7.2). Cells were incubated with hSulf-1 and CDK4 antibodies at 37°C overnight, washed with 0.1 M PBS (pH 7.2) and incubated as appropriate with FITC-conjugated anti-rabbit IgG and TRITC-conjugated anti-mouse IgG, respectively, at room temperature for 1 h. The expression and localization of hSulf-1 and CDK4 were observed using a confocal laser scanning microscope. (**D**, **E**) The expressions of AKT and CDK4 were silenced with shRNA vectors, Western blotting was performed with the same procedure as Figure 3A and 3B; ***P < 0.001 compared with the negative control group.

To verify the relationship between cell cycle regulation in melanoma cells by hSulf-1 and the activity of cell signaling pathways, we overexpressed hSulf-1 in the melanoma cell lines M21 and A375 via adenovirus infection and then examined changes in the protein levels of the signaling molecule protein kinase B (AKT) and the cell cycle regulator cyclin-dependent kinase 4 (CDK4). The results showed that compared with the negative control group, the phosphorylated AKT (p-AKT) was significantly reduced in the M21-hSulf1 and A375-hSulf1 cells, while the total AKT (t-AKT) and CDK4 contents did not change (Figure 3A). This result indicated that hSulf-1 can reduce the phosphorylation of AKT kinase. We further extracted nuclear and cytoplasmic proteins for western blotting and found that cytoplasmic CDK4 increased but nuclear CDK4 decreased in the M21-hSulf1 and A375-hSulf1 cells (Figure 3B). Confocal microscopy also showed that CDK4 was significantly reduced in the nucleus in the presence of ectopic hSulf-1 expression (Figure 3C). This result suggested that hSulf-1 expression can restrict the nuclear import of CDK4. We thus used pGenesil-shAKT to knock down AKT expression in the M21 and A375 parental cells, and we found that CDK4 nuclear import was suppressed, just as in the M21-hSulf1 and A375-hSulf1 cells (Figure 3D). This result indicated that the hSulf-1-induced change in CDK4 subcellular localization is related to AKT activity. We then used pGV102-shCDK4 to knock down CDK4 expression in the M21 and A375 cells. These cells showed no significant changes in the expression of hSulf-1 and the phosphorylation of AKT (Figure 3E).

### Expression of hSulf-1 inhibits melanoma xenograft growth in nude mice

**Figure 4:**
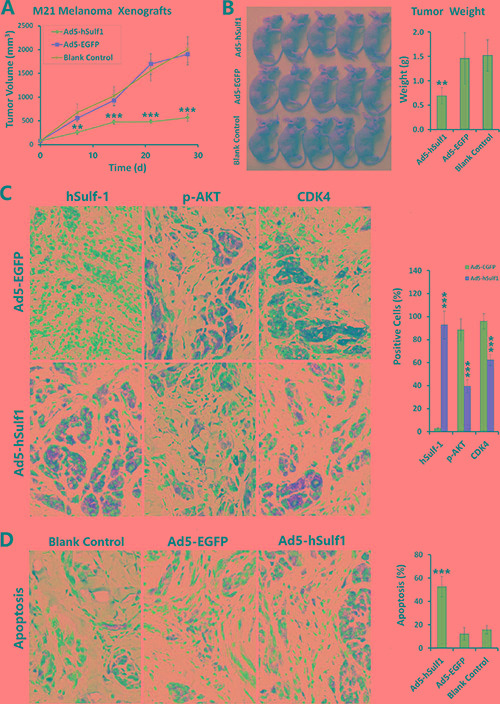
Expression of hSulf-1 inhibits melanoma xenograft growth in nude mice (**A**) M21 cells were used to establish xenograft model in nude mice, at 1 × 10^5^ cells/100 μl/animal. Nine days after inoculation, the mice were randomly divided into 3 groups (Ad5-hSulf1, Ad5-EGFP and control groups; *n* = 5 in every group). Ad5-hSulf1 and Ad5-EGFP were given daily multi-point injections in tumors at 1 × 10^8^ pfu/100 μl/animal for 10 continuous days. Control mice received saline injections of 100 μl per animal per day. Tumor sizes were measured weekly and tumor volumes were calculated as “maximum diameter × minimum diameter^2^ × 0.5”; **P < 0.01 and ***P < 0.001 compared with the Ad5-EGFP control group at the corresponding timepoint. (**B**) After the experiment was terminated, mice were sacrificed and tumors were collected and weighed; **P < 0.01 compared with the Ad5-EGFP control group. (**C**) The tumor specimens were fixed in 10% neutral buffered formalin, embedded in paraffin and sectioned. Immunohistochemistry was used to determine the expressions of hSulf-1, p-AKT and CDK4. Hematoxylin staining was used to show nuclei with blue color. Diaminobenzidine (DAB) staining was used to show the positive expressions of factors with brown color; ***P < 0.001 compared with the Ad5-EGFP control group; original magnification: 200×. (**D**) TUNEL assay was performed to detect the ratio of apoptotic cells. Hematoxylin staining was used to show nuclei with blue color. DAB staining was used to show the positive expressions of factors with brown color; ***P < 0.001 compared with the Ad5-EGFP control group. The percentages of positive cells on each slide were determined by counting within 5 high-power fields; ***P < 0.001 compared with the Ad5-EGFP control group.

By xenografting M21 melanoma cells in nude mice, we observed the effects of adenovirus-mediated hSulf-1 expression on growth speed of tumors and AKT signaling pathway activity of tumor cells. We observed that ectopic hSulf 1-expression could significantly reduce the growth speed of transplanted tumors. On the 7th day after treatment, the tumor volumes in the Ad5-hSulf1 treatment mice were significantly lower than those of the negative control group (*P* < 0.01). By day 21, the difference was very significant (*P* < 0.001). In contrast, tumors treated with the Ad5-EGFP control virus did not show any tumor growth inhibition throughout the experiment (Figure 4A).

The experiment ended after 28 days of treatment, at which time the tumors were weighed. The tumors that had been treated with Ad5-hSulf1 were significantly lighter than the tumors treated with the Ad5-EGFP control virus and the tumors in the negative control group (*P* < 0.01; Figure 4B). We prepared tumor tissue sections and used immunohistochemistry to detect hSulf-1 expression, CDK4 expression and AKT phosphorylation levels. We found that with hSulf-1 expression, AKT phosphorylation in melanoma cells was significantly reduced, as were nuclear CDK4 levels (Figure 4C). We used the transferase-mediated deoxyuridine triphosphate-biotin nick end labeling (TUNEL) method to detect apoptotic cells and found that the percentage of apoptotic tumor cells in the Ad5-hSulf1 group was significantly higher than in the Ad5-EGFP control group or the blank control group (*P* < 0.001; Figure 4D).

## DISCUSSION

The hSulf-1 is a tumor suppressor gene that is often in an inactivated state in human malignancies. The biological function of hSulf-1 is to desulfate cell surface HSPG via the hydrolysis of 6-O-bond glucosamine sulfate in the highly sulfated sugar chains of HSPG [12]. Sulfation of HSPG is critical for interactions between extracellular growth factors and their receptor tyrosine kinases [13, 14]. Accordingly, desulfation of HSPG caused by hSulf-1 is capable of inhibiting the signaling pathways of various growth factors, including those of epidermal growth factor (EGF), fibroblast growth factor (FGF) and vascular endothelial growth factor (VEGF). As such, hSulf-1 plays important roles in the regulation of cell differentiation, proliferation, adhesion, tissue repair, inflammation, angiogenesis and tumorigenesis [2, 5, 10]. Studies have shown that introducing exogenous hSulf-1 into tumor cells reduced their proliferation rate, increased apoptosis, increased the sensitivity of tumor cells to chemotherapeutic drugs, and inhibited tumor cell proliferation, metastasis and interstitial vascularization.

The mechanism of HSPG action is very complicated. Studies have reported that heparin-binding EGF (HB-EGF) can bind to EGFR when mediated by sulfated HSPG, thereby promoting sustained phosphorylation of EGFR on Tyr1068 and Tyr992 residues and activating the downstream AKT and ERK signaling pathways to promote cancer cell proliferation and migration [15]. hSulf-1 can inhibit the formation of the HB-EGF/HSPG/EGFR complex and thus exert anti-tumor activity [16]. Activation of the HGF/c-Met signaling pathway is a common phenomenon in tumors that can trigger changes in cellular phenotypes to enhance cell invasion and metastasis [17]. Expression of hSulf-1 can also reduce HGF-mediated PI3K/AKT pathway activity, thereby inhibiting tumor growth and metastasis [18]. hSulf-1 expression is widely down-regulated in many tumor tissues and tumor cell lines, and it provides an ideal biological target for cancer treatment. Based on the mechanism of hSulf-1 action, we hypothesized that hSulf-1 expression, by inhibiting the sulfation of HSPG on the surface of cancer cells, can block the binding of various growth factors to their receptors and thus broadly inhibit signaling pathways implicated in cancer cell proliferation and metastasis. Such activity would serve to improve the efficacy of cancer treatment.

Melanoma is one of most aggressive malignant diseases with high mortality and poor prognosis. Surgery is the first choice for treatment of early melanoma, but majority of patients present at later stage when they were diagnosed and lost surgical opptunity [19]. Treatment of melanoma with chemotherapy remains unsatisfactory. Studies on molecular mechanisms clarified that many signal transduction pathway are involed in regulation of melanoma biological behaviours, for example, activation of both ERK1/2 and AKT signaling pathways can promote melanoma cell viability, motility, and anchorage-independent growth, which is valuable for research and development of target therapeutic drugs [20]. Although it is well known that the sulfated HSPG activates the activities of many growth factor receptor pathways and promotes tumor progression in various cancers, there is no systematic study on the relationship between melanoma and HSPG or hSulf-1 [21]. In this study, we found that the melanoma cell line M21 did not express hSulf-1, while A375 cells weakly expressed hSulf-1. The overexpression of hSulf-1 in M21 and A375 cells inhibited cell proliferation, and cells lost their response to a pro-proliferative growth factor bFGF. These results strongly implicate hSulf-1 in the regulation of proliferation in melanoma cells. hSulf-1 overexpression was not only capable of inducing apoptosis in melanoma cells, but it also blocked cell cycle progression, thereby exerting anti-tumor activity. Further study revealed that the hSulf-1-induced changes in the biological behaviors of melanoma cells are closely associated with reduced AKT phosphorylation and reduced CDK4 nuclear import. p-AKT levels were significantly reduced in melanoma cells expressing hSulf-1, and cytoplasmic CDK4 levels increased while nuclear CDK4 levels decreased. Knocking down AKT in the parental M21 and A375 melanoma cells also appeared to inhibit CDK4 nuclear import, suggesting that the hSulf-1-induced changes in CDK4 subcellular localization are related to AKT. Cell proliferation and cell cycle progression are related to the phosphorylation and activation of a series of CDK molecules. At the G1/S cell cycle checkpoint, CDK4 is an important positive regulator [22]. CDK4 must translocate from the cytoplasm to the nucleus to exert its function in cell cycle regulation. With hSulf-1-induced decreases in AKT pathway activity and CDK4 nuclear import, CDK4 function is inhibited. This may be an important reason for why hSulf-1 expression induces melanoma cell cycle arrest.

In xenograft experiments with M21 melanoma cells in nude mice, we demonstrated that hSulf-1 expression retarded the growth rate of xenografted tumors. Immunohistochemistry results showed that AKT phosphorylation in tumor cells was decreased and that nuclear CDK4 levels were reduced. Our animal experiments demonstrated that the use of hSulf-1 to inhibit cell surface HSPG sulfation as an intervention strategy is an effective way to treat melanoma. In summary, the inactivation of hSulf-1 in melanoma cells furnishes an ideal molecular target for treating melanoma. hSulf-1 exerts its anti-tumor effects by inhibiting cell surface HSPG sulfation, thus lowering downstream AKT signaling pathway activity and decreasing the nuclear import of the cell cycle regulator CDK4.

## MATERIALS AND METHODS

### Vector construction

An adenovirus vector Ad5-hSulf1, which bears the gene that encodes hSulf-1, and a control adenovirus vector Ad5-EGFP were successfully constructed in previous studies and were experimentally confirmed [2, 5]. An shRNA vector for silencing AKT (pGenesil-shAKT) was constructed by Genesil Biotechnology, Wuhan, China. It contains a 19-nt DNA sequence (5′-gactacctgcactcggaga-3′) that targets base pairs 1338–1356 of the Akt1 gene (NM_005163). An shRNA vector for silencing CDK4 (pGV102-shCDK4) was constructed by Shanghai Jikai GeneChem Technology Co., Ltd. It contains a 19-nt DNA sequence (5′-tcgttcaccgagatctgaa-3′) that targets base pairs 699–717 of the CDK4 gene (NM_000075). A control shRNA vector (pCtrl-shRNA, 5′-gacttcataaggcgcatgc-3′) was provided by Genesil Biotechnology, Wuhan, China.

### Gene expression modulation in melanoma cell lines

The melanoma cell lines M21 and A375 were obtained from the Shanghai Institute of Cell Biology, Chinese Academy of Sciences. Cells were cultured under the conditions recommended by the supplier. Melanoma cells were infected with adenovirus Ad5-hSulf1 at a multiplicity of infection (MOI) of 100 pfu/cell. Cell lines positively expressing hSulf-1 were established as M21-hSulf1 and A375-hSulf1. Melanoma cells were also transfected with the pGenesil-shAKT and pGV102-shCDK4 vectors. Cells were plated into 6-well plates at 1 × 10^6^ cells per well, and each well received a final vector concentration of 20 μg/ml. Cells were then cultured for 48 h. A portion of the cells was harvested for extraction of total protein and gene expression analysis; the remaining cells were used to characterize cellular behaviors.

### Immunoblotting

Total proteins were extracted with protein extraction reagents (Pierce Biotechnology, Inc., Rockford, IL, USA) from melanoma cell lines M21 and A375 and from their derivative cell lines produced by the above-mentioned plasmid transfection or viral infection. Nuclear and cytoplasmic proteins were extracted using nuclear and cytoplasmic extraction reagents, respectively (Pierce Biotechnology, Rockford, Illinois, USA). Western blotting assays were used to determine the protein levels of hSulf-1 (rabbit anti-human sulfatase 1, Abcam Company Ltd., Shanghai, China), total AKT (t-AKT), phosphorylated AKT (p-AKT, mouse anti-AKT and rabbit anti-phosphorylated AKT, Cell Signaling Technology Inc., Shanghai, China) and CDK4 (mouse anti-CDK4, Cell Signaling Technology) [23].

### Confocal immunofluorescence microscopy

The melanoma M21 cells and their transfected or infected derivatives were cultured in Lab-Tek chambers (Electron Microscopy Sciences, Hatfield, Pennsylvania, USA) at 1 × 10^4^ cells per well. After 24 h, cells were fixed for 30 min in 4% formaldehyde and washed with 0.1 M PBS (pH 7.2). Cells were incubated with hSulf-1 and CDK4 antibodies (working concentration, 1:500) at 37°C overnight, washed with 0.1 M PBS (pH 7.2), and incubated as appropriate with FITC-conjugated anti-rabbit IgG and TRITC-conjugated anti-mouse IgG (Santa Cruz Biotechnology, Inc., Santa Cruz, CA) at room temperature for 1 h. hSulf-1 and CDK4 expression and localization were observed using a confocal laser scanning microscope (Zeiss LSM510, Carl Zeiss, Germany).

### Cell proliferation assay

The melanoma cell lines M21 and A375 and their transfected or infected derivatives were seeded into 96-well plates (1 × 10^4^ cells/well). Once the cells attached, the growth factor bFGF (Sigma-Aldrich, Shanghai, China) was added into the wells at a final concentration of 20 ng/ml. After 24 h, 48 h, 72 h, and 96 h, proliferation was examined using the Cell Counting Kit-8 (CCK-8) (Dojindo Molecular Technologies, Inc., Shanghai, China) according to the manufacturer's instructions. Each time point corresponded to 8 replicate wells.

### Detection of cell cycle and apoptosis

The melanoma cell lines M21 and A375 and their transfected or infected derivatives were cultured for 48 h. After the cells were collected, a portion of the cells was fixed with ice-cold 75% ethanol and incubated in a 4°C refrigerator overnight. Cells were washed twice in PBS and then incubated with a propidium iodide (PI) staining mixture containing RNase for 30 min in dark. The cell cycle status was detected by flow cytometry. The remainder of the cells was stained with Annexin V/PI. Apoptosis was detected with a flow cytometer (FACS420, BD Biosciences, San Jose, CA, USA).

### Xenograft model experiments

Twenty healthy, purebred 4-week-old male BALB/c mice were provided by the Shanghai SLAC Experimental Animal Center of the Chinese Academy of Sciences. M21 melanoma cells were cultured to logarithmic growth phase and then subcutaneously injected underneath the right arm at 1 × 10^5^ cells/100 μl/animal. Nine days after inoculation, the rate of tumor onsite was 100%. The average maximal diameter of the xenograft tumors was 0.49 ± 0.12 cm. After excluding the mice with the 2 biggest tumors and the 3 smallest tumors, the remaining 15 mice were randomly divided into 3 groups (Ad5-hSulf1, Ad5-EGFP and a control group). The Ad5-hSulf1 and Ad5-EGFP groups were given daily multi-point injections of the corresponding adenovirus in the tumors at 1 × 10^8^ pfu/100 μl/animal for 10 continuous days, for a total dose of 1 × 10^9^ pfu/animal. Control mice received saline injections of 100 μl per animal per day. After treatment, tumor sizes were measured weekly; tumor volumes were calculated as “maximum diameter × minimum diameter^2^ × 0.5”. When the average volume of tumors in any group exceeded 2,000 mm^3^, the experiment was terminated as per the regulations of the Animal Ethics Committee of the Second Military Medical University. At the end of the observation period, mice were anesthetized and sacrificed. The tumor specimens were weighed, fixed in 10% neutral buffered formalin, embedded in paraffin and sectioned. Immunohistochemistry was used to determine the expression levels of hSulf-1, p-AKT and CDK4. TUNEL (Fuzhou Maixin Biotechnology Development Co., Fuzhou, China) was used to detect the ratio of apoptotic cells. Ratios of positive cells on each slide were determined by counting 5 high-power fields.

### Statistical analyses

The experimental data from 3 times of independent *in vitro* experiments, as well as the *in vivo* experimental data from 5 mice per group, were presented as ‘mean ± standard deviation (SD)’, and analyzed by one-way analysis of variance (ANOVA). When the data were statistically different among the multiple groups, the SNK-q test was used to conduct the multiple comparisons. The statistical analysis software package PASW Statistics 18 was used. *P* values less than 0.05 were considered statistically significant.
